# Behçet Disease

**DOI:** 10.5334/jbsr.2949

**Published:** 2022-11-09

**Authors:** Kelly Di Dier, Benjamin Leenknegt, Marc Lemmerling

**Affiliations:** 1 AZ Sint-Lucas, Ghent, BE

**Keywords:** Behçet disease, vasculitis, aortitis, inferior caval vein thrombosis, Budd Chiari syndrome, computed tomography, ultrasound

## Abstract

**Teaching Point:** Although Behçet disease is a multisystemic and chronic vasculitis, it can be superimposed with a variety of acute vasculitis.

## Case History

**Figure 1:**
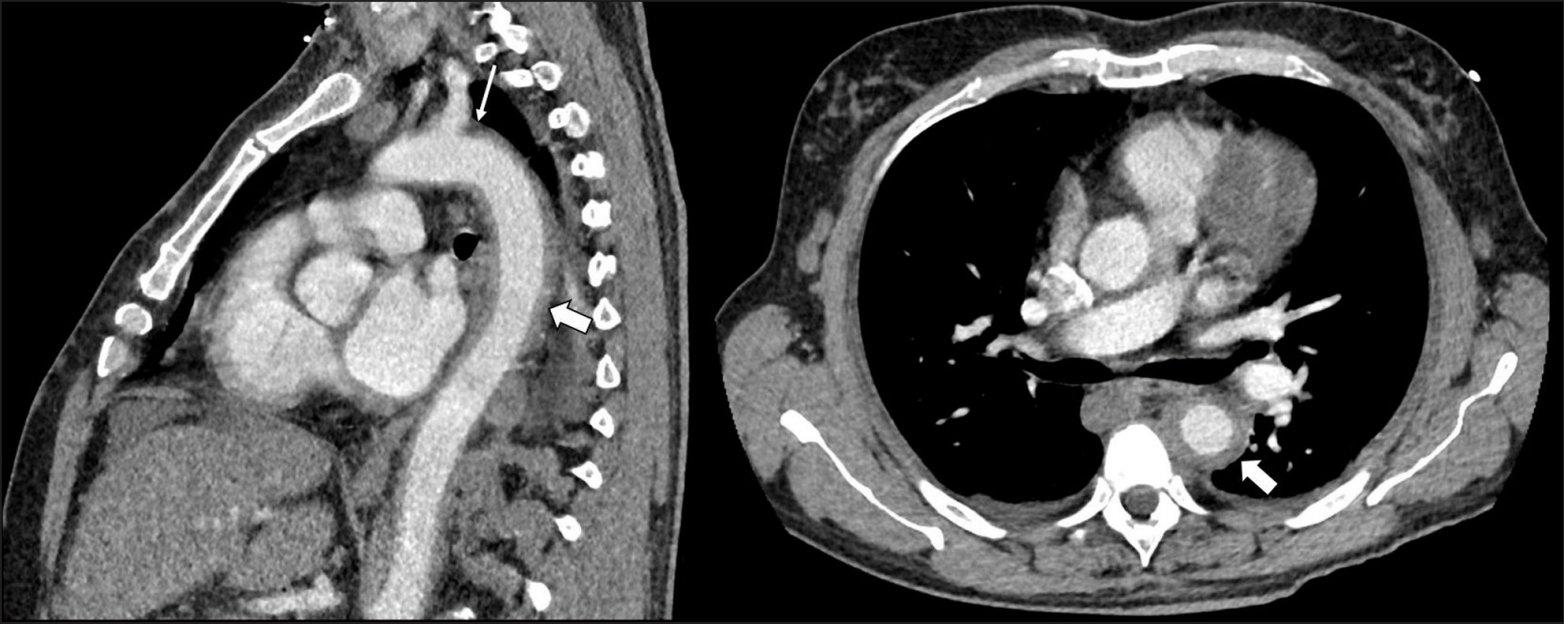


**Figure 2:**
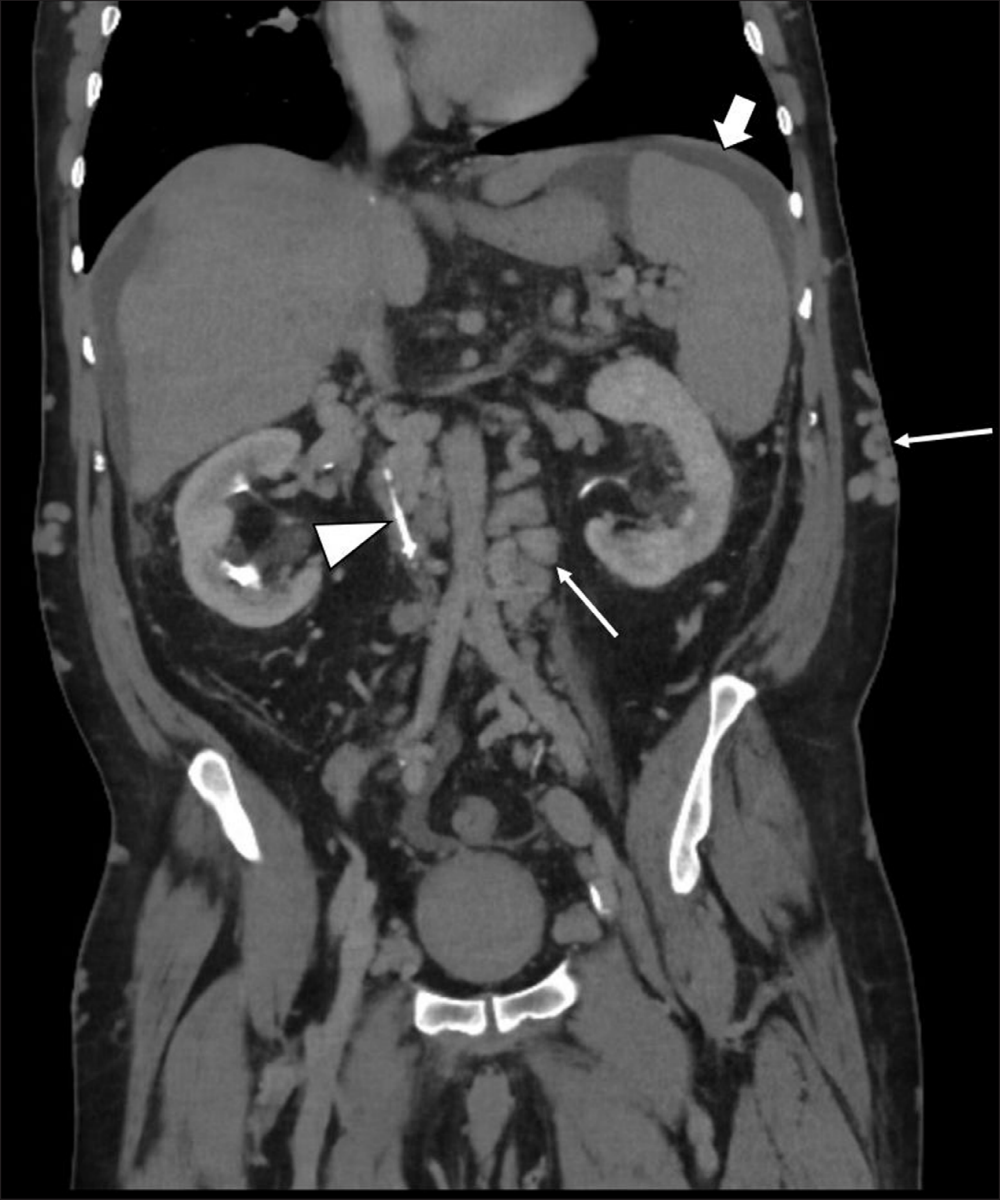


**Figure 3:**
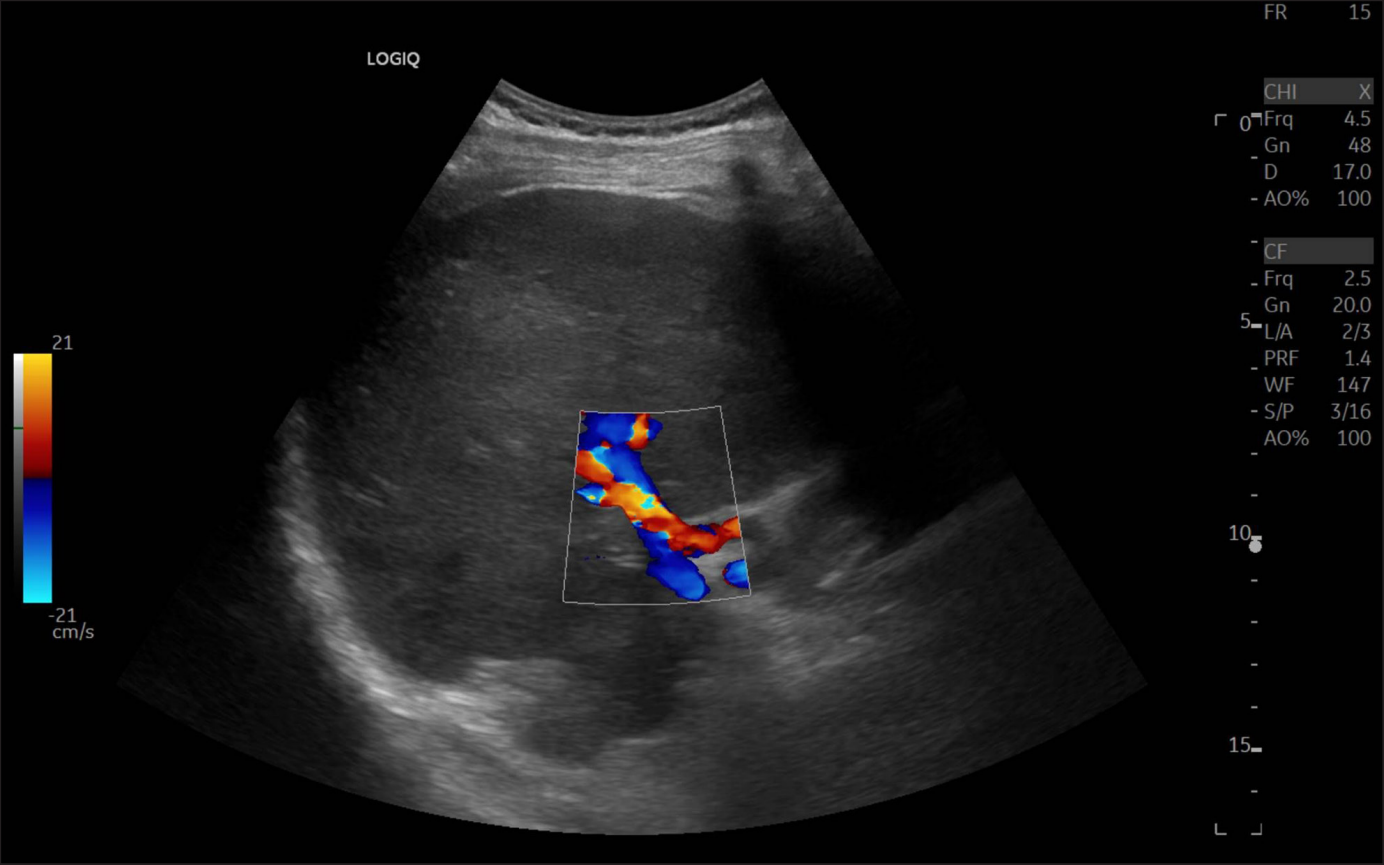


A 55-year-old Middle Eastern male presented to the emergency department with fever and a constricting chest pain. He was known to have multisystemic Behçet disease and had multiple severe skin ulcerations. A computed tomography (CT) pulmonary angiography was performed to exclude pulmonary embolism. On the CT, a circumferential rim of inflammation around the proximal left subclavian artery (Figure 1, small arrow), the aortic arch and the descending aorta (Figure 1, arrow) was observed, consistent with the diagnosis of aortitis. Due to the presence of ascites in the upper abdomen, a CT of the abdomen was performed, which revealed a chronic calcified thrombosis of the inferior caval vein (Figure 2, arrowhead) and an occlusion of the hepatic veins. In addition to ascites (Figure 2, arrow), other features of portal hypertension were an extensive abdominal venous collateral circulation (Figure 2, small arrows), portal flow inversion and hepatic artery hypertrophy on duplex ultrasound (Figure 3, blue and red flow signal). The combination of hepatic vein occlusion and signs of portal hypertension favours the diagnosis of Budd Chiari syndrome.

## Comment

Behçet disease is a chronic and multisystemic inflammatory vasculitis of unknown origin. It is more common in the Mediterranean and East-Asian population. The disease most often presents in the second or third decade and has a male predominance. The gastro-intestinal tract, the skin, the cardiovascular, neurologic and thoracic systems are most commonly involved [1].

Different intrathoracic structures can be affected. Aneurysms or thromboembolic events will predominantly occur in the aorta, the subclavian arteries and the coronary arteries. Aortitis is less frequent and may be an acute or chronic manifestation [1]. In our case, acute aortitis presented as an inflammatory wall thickening of the aorta.

Thromboembolism of the visceral veins and arteries also may occur. Occlusion of the inferior caval vein and/or the hepatic veins may compromise the hepatic outflow and result in Budd Chiari syndrome. Although rare in general, Budd Chiari syndrome is more frequently observed in patients with Behçet disease [1]. A cascade of hepatic congestion, increasing liver stiffness, portal hypertension and portal flow reversal ends in intra-abdominal venous collateralisation and ascites, as is present in our case.

In conclusion, Behçet disease is a chronic and multisystemic inflammatory vasculitis which can have acute thoracic and abdominal manifestations.
